# The Interplay between Metabolism, PPAR Signaling Pathway, and Cancer

**DOI:** 10.1155/2017/1830626

**Published:** 2017-04-26

**Authors:** Daniele Fanale, Valeria Amodeo, Stefano Caruso

**Affiliations:** ^1^Department of Surgical, Oncological and Oral Sciences, Section of Medical Oncology, University of Palermo, 90127 Palermo, Italy; ^2^Samantha Dickson Brain Cancer Unit, UCL Cancer Institute, University College London, London WC1E 6DD, UK; ^3^Génomique Fonctionnelle des Tumeurs Solides, INSERM, UMR 1162, 75010 Paris, France

The peroxisome proliferator-activated receptors (PPARs) belong to the ligand-inducible nuclear hormone receptor superfamily including three major members: PPAR*α* (also called NR1C1), PPAR*β*/*δ* (also called NR1C2), and PPAR*γ* (also called NR1C3). Despite several similarities, each PPAR isoform shows specific functions likely due to different biochemical properties, variable tissue distribution, and differential responses to different ligands. PPAR transcriptional activity can be modulated through a nongenomic cross talk with phosphatases and kinases, including ERK1/2, p38-MAPK, PKA, PKC, AMPK, and GSK3. PPARs can form heterodimers with retinoid X receptor (RXR) modulating the expression of genes involved in lipid metabolism, adipogenesis, maintenance of metabolic homeostasis, and inflammation and inducing also anticancer effects in a variety of human tumors. Although all PPAR isoforms are implicated in several metabolic syndromes, PPAR*γ* seems to be mostly involved in tumorigenesis regulation via activation of different pathways. Therefore, the modulation of PPAR signaling pathways could be a potential novel strategy to inhibit carcinogenesis and tumor progression. PPAR*γ* can be activated by natural ligands, such as fatty acids and their derivatives, as well as synthetic ligands, such as thiazolidinediones (TZDs), including ciglitazone, rosiglitazone, troglitazone, and pioglitazone. TZDs have been shown to be a class of drugs with potent insulin-sensitizing activity used to improve lipid and glucose metabolism in obesity and type 2 diabetes via PPAR. Since accumulating evidence highlighted the role of PPAR signaling in carcinogenesis, type 2 diabetes, and other metabolic disorders, such as obesity, understanding the potential molecular mechanisms underlying the interplay between metabolism, PPAR signaling, and cancer may represent an interesting research field for the development of novel strategies useful for the prevention and treatment of cancer. Since there exists a strong correlation between obesity and diabetes and both have been also shown to increase cancer risk, the identification of the molecular mechanisms by which PPAR modulates these events may help us to better understand how the diet may affect the cancer susceptibility.

In this special issue we have assembled different studies describing possible intracellular pathways involved in these processes, in order to increase the current knowledge about the correlation between metabolic syndromes and cancer via activation of different PPAR signaling pathways. In particular, our endpoint was to provide an overview about the potential roles of PPARs in modulating the cancer risk induced by metabolic disorders such as diabetes and obesity. Here, we gathered 1 original research paper and 9 review articles that describe and discuss the various functions of PPARs in the context of several metabolic alterations associated with different types of cancer.

In our review article titled* “*Potential Role of ANGPTL4 in the Cross Talk between Metabolism and Cancer through PPAR Signaling Pathway,*”* we speculated and discussed the potential role of ANGPTL4 as key player in mediating the cross talk between metabolic syndromes, such as diabetes and obesity, and cancer through regulation of its expression by PPARs. Indeed, since ANGPTL4 is involved in several metabolic conditions, both physiological and pathological, including angiogenesis, glucose homoeostasis, lipid metabolism, and tumorigenesis, we hypothesized that its transcriptional regulation by PPARs could represent a gateway between obesity, insulin sensitivity, and cancer.

The review article by S. G. Vitale et al.* “*Peroxisome Proliferator-Activated Receptor Modulation during Metabolic Diseases and Cancers: Master and Minions*”* provided an overview on the different roles of PPARs in control of the expression of genes involved in energy homeostasis, cell proliferation, and apoptosis, discussing the involvement of these nuclear receptors in tumorigenesis and development of metabolic diseases.

B. B. Merchan and collaborators in their review article titled* “*Commonalities in the Association between PPARG and Vitamin D Related with Obesity and Carcinogenesis*”* have focused on the recent advances that led to hypothesizing a possible cross talk between PPARG, Vitamin D system, obesity, and cancer, suggesting a close cooperation between the vitamin D/VDR system and PPARG signaling in order to maintain a correct metabolic homeostasis. Interestingly, the authors reported several studies describing how the shortage of Vitamin D and decrease in PPARG levels may be involved in obesity and cancer development.

In another manuscript titled* “*PPAR Gamma in Neuroblastoma: The Translational Perspectives of Hypoglycemic Drugs*”* S. Vella et al., by reviewing literature data, discussed the potential beneficial effects of hypoglycemic drugs, such as thiazolidinediones and metformin, in treatment of neuroblastoma patients.

A noteworthy contribution to this special issue was the review article titled* “*PPAR*δ* as a Metabolic Initiator of Mammary Neoplasia and Immune Tolerance*”* by R. I. Glazer describing the involvement of PPAR*δ* in the initiation and promotion of mammary tumorigenesis through its pivotal role in regulating metabolism, inflammation, and immune tolerance.

An interesting review article by T. Mello and colleagues titled* “*PPARs and Mitochondrial Metabolism: From NAFLD to HCC*”* described the functions of PPARs in the modulation of liver mitochondrial metabolism during the progression from nonalcoholic fatty liver disease to hepatocellular carcinoma, hypothesizing the possibility, in future, of using new therapeutic approaches able to selectively target the fuel requirements of HCC.

In the review article titled* “*MicroRNAs-Dependent Regulation of PPARs in Metabolic Diseases and Cancers,*”* D. Portius et al. stressed out the miRNA-mediated regulation of PPARs in the context of metabolic disorders, inflammation, and cancer. Moreover, the authors evaluated the reciprocal control of miRNA expression by PPARs, to finally speculate about the therapeutic potential of modulating PPAR expression/activity by pharmacological compounds targeting miRNAs.

Instead, S. P. Lakshmi et al. provided a review article titled* “*PPAR Agonists for the Prevention and Treatment of Lung Cancer,*”* where authors described the role of PPAR*α*, PPAR*β*/*δ*, and PPAR*γ* in lung cancer pathogenesis and dissected the current literature on the multifaceted effects of PPAR agonists in lung cancer treatment. Finally, the authors discussed how PPAR ligands may be used in the context of novel therapeutic strategies against this disease.

In* “*Deciphering the Roles of Thiazolidinediones and PPAR*γ* in Bladder Cancer*”* M. Chiu and colleagues discussed how some thiazolidinediones, synthetic ligands of PPAR*γ* used in the treatment of diabetes mellitus type 2, could significantly increase the risk of developing bladder cancer.

Lastly, A. A. Koronowicz and collaborators in their research paper titled* “*Fatty Acids of CLA-Enriched Egg Yolks Can Induce Transcriptional Activation of Peroxisome Proliferator-Activated Receptors in MCF-7 Breast Cancer Cells*”* investigated the effects in vitro of fatty acids from CLA-enriched egg yolks (EFA-CLA) acting as potential ligands for PPAR receptors in breast cancer cell line MCF-7. The authors showed that PPAR-responsive genes can be regulated by EFA-CLA, leading to a reduction of tumor cell proliferation, with a greater influence by EFA-CLA treatment compared to nonenriched FAs or single synthetic CLA isomers.

In conclusion, understanding the different roles of PPARs in the cross talk between metabolic syndromes, such as diabetes and obesity, and cancer could help us, in future, to identify novel potential therapeutic targets involved in the cancer development and different cancer-related metabolic syndromes. This special issue, thanks to the interesting contributions by various authors, attempts to provide a small overview of the studies present in literature regarding the involvement of PPAR Signaling Pathway in carcinogenesis and metabolic alterations, enriching the current knowledge in the field and opening up new roads towards multidisciplinary approaches that promote the interaction between basic research and clinical research.

*Daniele Fanale* *Valeria Amodeo* *Stefano Caruso*

